# Computational Methods for the Analysis of Array Comparative Genomic Hybridization

**Published:** 2007-02-10

**Authors:** Raj Chari, William W. Lockwood, Wan L. Lam

**Affiliations:** 1 Cancer Genetics and Developmental Biology, British Columbia Cancer Research Centre, Vancouver BC, Canada V5Z 1L3;; 2 These authors contributed equally to this work

**Keywords:** array CGH, microarray, cancer genome, software, bioinformatics, alteration detection

## Abstract

Array comparative genomic hybridization (array CGH) is a technique for assaying the copy number status of cancer genomes. The widespread use of this technology has lead to a rapid accumulation of high throughput data, which in turn has prompted the development of computational strategies for the analysis of array CGH data. Here we explain the principles behind array image processing, data visualization and genomic profile analysis, review currently available software packages, and raise considerations for future software development.

## Background

**Figure 1.**
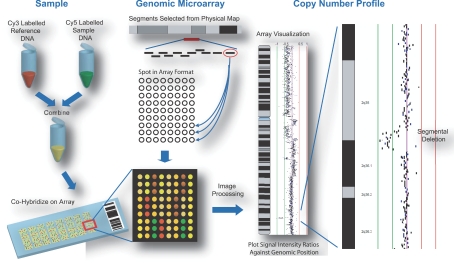
Generation of array comparative genomic hybridization profiles. Tumor and normal reference DNA are differentially labeled with cyanine-5 and cyanine-3 respectively and competitively hybridized to a genomic microarray. The array consists of DNA targets selected to span chromosome regions or the entire genome. These targets are typically spotted in replica. The ratio of the two fluorescence signal intensities reflects the relative copy number at that target. The ratio for each spot is plotted against its corresponding position in the human genome to generate a copy number profile.

Segmental deletion and duplication of chromosomal regions have been associated with both constitutional diseases and somatic alterations in cancer (Inazawa et al. 2004; Lockwood et al. 2006; Oostlander et al. 2004; Vissers et al. 2005). Recent studies have demonstrated that large scale copy number variations exist in the human population (Conrad et al. 2006; de Vries et al. 2005; Hinds et al. 2006; Iafrate et al. 2004; McCarroll et al. 2006; Sebat et al. 2004; Tuzun et al. 2005). Array comparative genomic hybridization (array CGH) is a method designed for identifying genomic regions with copy number aberration (Pinkel et al. 1998; Solinas Toldo et al. 1997). In this method, DNA from both reference and test genomes are differentially labeled with fluorescent dyes and competitively hybridized to DNA targets arrayed on a glass slide (Fig. 1). The hybridized slide is then scanned and the resulting signal intensity ratio at each DNA target reflects the copy number status of the DNA segment. By referring the segment to its corresponding position on the human genome map, the genes affected by copy number alteration can be identified (Fiegler et al. 2003; Ishkanian et al. 2004; Snijders et al. 2001). Numerous advances in array CGH technology have been made since its development in the mid 1990s with increased genome coverage and target density, improving resolution and sensitivity of detection. The majority of array CGH platforms use either oligonucleotide (oligo) or large insert clone (LIC) DNA targets (Davies et al. 2005). Oligos are short DNA fragments of approximately 21–60 nucleotides in length whereas LICs are typically bacterial artificial chromosome (BAC) clones which are ~100 kb in size. Historically, arrays were designed to cover specific chromosomes (Buckley et al. 2002; Buckley et al. 2005), chromosome arms (Coe et al. 2005; Garnis et al. 2003; Henderson et al. 2005) or selected regions of the genome implicated in disease (Albertson et al. 2000; Schwaenen et al. 2004). In contrast, genome wide arrays that sample copy number status of loci at megabase intervals have facilitated rapid survey for regions of loss and gain (Fiegler et al. 2003; Greshock et al. 2004; Snijders et al. 2001). Alternatively, cDNA microarrays, initially designed for gene expression profiling, have been used to assess copy number status of coding regions (Pollack et al. 1999; Squire et al. 2003). The development of high density arrays consisting of tens of thousands of DNA targets spanning the entire human genome has enabled precision mapping of the boundaries of genetic alterations throughout the genome in a single experiment (Barrett et al. 2004; Bignell et al. 2004; Ishkanian et al. 2004; Selzer et al. 2005; Zhao et al. 2004).

The production and use of these high density arrays relies not only on technical precision in array synthesis but also computational platforms tailored to the imaging, mapping, and analysis of replica sets of tens of thousands of DNA targets with spot signals in a narrow dynamic range. This article describes the principles behind visualization and analysis of whole genome array CGH data and reviews the software currently available.

## Analysis of Array CGH Data

**Figure 2.**
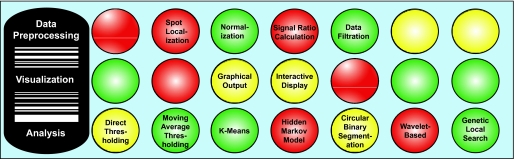
Principles of array CGH analysis. The process is grouped into three general functions: data preprocessing, visualization, and detection of segmental alterations, in no particular order. Methodologies for each function are indicated in a horizontal manner.

Array CGH software applications can be classified according to three general functions: data preprocessing, visualization, and analysis (Fig. 2). Some software programs are specific to a particular function while others perform multiple tasks. The following section explains the principles and describes the methods for performing these functions.

### Data pre-processing

**Figure 3.**
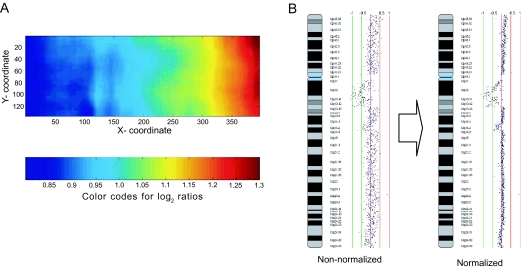
Normalization of array CGH data. **A**: A plot illustrating spatial bias across the microarray. **B**: The copy number profile of a chromosome before and after normalization. The removal of systematic biases improves the conformity of the profile.

Upon completion of an array CGH experiment, the microarray slide is scanned in two channels to generate high resolution fluorescence images corresponding to the two cyanine dyes. The localization of spots on the array is a semi-automatic process supported by “spot finding” functions, available in most microarray scanner software packages and custom packages (Jain et al. 2002). The signal intensity for each spot is quantified for each channel. However, image normalization is critical to improving detection sensitivity of copy number alterations, as a single copy loss would only reduce the signal by 50% resulting in a 1:2 signal ratio, and a single copy gain would result in a 3:2 ratio. (These shifts in ratios are subtle compared to gene expression changes.) In tumor samples, these ratios are further dampened by tissue heterogeneity with a mixed population of normal and cancer cells (Garnis et al. 2005). Therefore, before signal ratio can be deduced, the intensities of the two images need to be balanced and systematic biases influencing measurements need to be removed (Fig. 3). Intensity bias (different intensities for the dye channels), spatial bias (the location of DNA target on the array), plate bias (source plate of the target DNA spotted) and background bias (the contribution of background fluorescence to spot signal intensity) are factors that have been shown to affect signal intensity ratio in high density array CGH experiments (Khojasteh et al. 2005).

### Data visualization

As replica spots are necessary to ensure experimental precision, arrays often contain multiple measurements of a DNA target. Therefore, basic operations are applied to determine the mean or median ratios of the replica, and the standard deviation for quality assessment and filtering.

**Figure 4.**
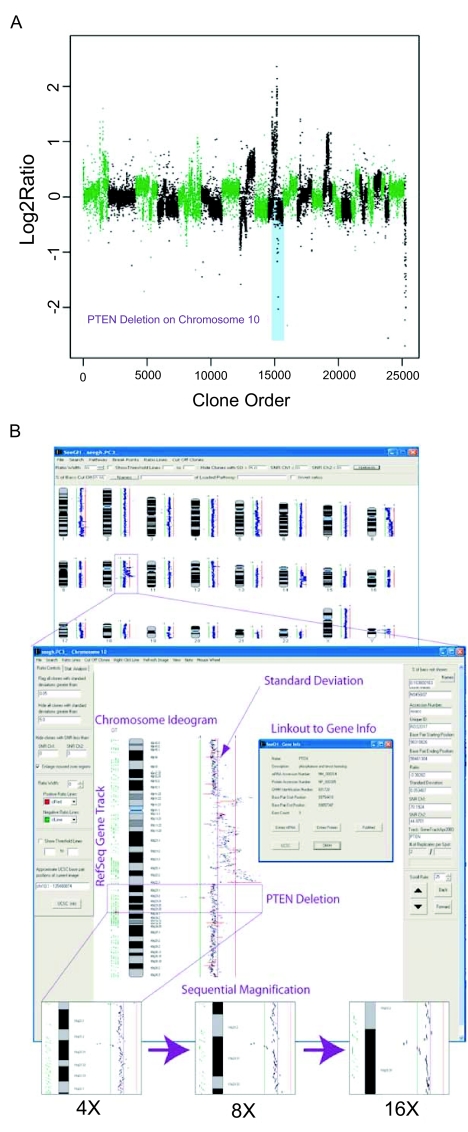
Visualization of array CGH data. **A**: A graphical representation of array CGH data. The chromosomes are alternately labeled in green and black. In this graph, log_2_ signal ratio for each clone is plotted against its chromosomal position ordered in series. *PTEN* deletion is highlighted in blue. **B**: Interactive display of the same data emphasizing the options to magnify selected chromosomes or chromosome segments, to display aligned gene annotation (gene track) and to link to external biological databases. The corresponding *PTEN* region in a) is indicated.

To display spot data in the context of genomic position, log_2_ signal ratio for each spot is plotted against its corresponding location in the human genome. Graphical representations and interactive display are the two main approaches used in visualization. Graphical representations are XY scatter plots, with the X axis representing the array elements in ordered chromosomal position—typically, the chromosomes are arranged in series—and the Y axis representing the corresponding log_2_ signal ratio. However, with arrays containing tens of thousands of DNA elements (high density arrays), the number of data points are too numerous to display on this scale (Fig. 4a). Interactive displays are designed for high density arrays allowing the sequential magnification of selected chromosomes and chromosome segments to visualize individual data points. Typically, ratio data is displayed in parallel to a chromosome ideogram. Advanced visualization software provide practical features, for example, displaying the actual segment length represented by the spotted element (as opposed to non-overlapping single points), displaying aligned gene annotation (gene track), providing immediate linkage to public databases such as Online Medelian Inheritance of Man (OMIM), NCBI Entrez and UCSC Genome Browser (Fig. 4b).

### Detection of segmental alterations

A variety of methods are used in the identification of segmental copy number alterations. Here we describe the principles behind the commonly used analytical approaches (Fig. 2).

### Direct thresholding

**Figure 5.**
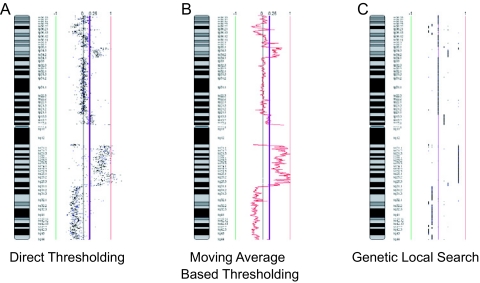
Analysis of array CGH data. Three of the methods described in the text for the detection of segmental alterations are illustrated. **A**) Direct thresholding, gains and losses are based on a theoretical ratio, in this case the indicated purple line, using the individual values for each clone on the array. **B**) Moving average based thresholding involves the calculation of the average ratio across a sliding window of clones prior to implementation of a threshold, indicated by the red line. The threshold line is indicated in purple. **C**) Genetic local search is an algorithm that partitions the data into segments and then “smooths” the data by calculating the average of all the data points within each segment. Smooth segments are indicated by black lines.

One of the simplest approaches for data analysis is by directly thresholding at a particular ratio. This methodology was very commonly used in early array CGH publications (Albertson et al. 2000; Garnis et al. 2004; Veltman et al. 2003). This threshold value can be defined in different ways. Ratio thresholds signify gains and losses based on a theoretical ratio of a single copy gain (3:2, log_2_ ratio of 0.585) and single copy loss, (1:2, log_2_ ratio of -1), albeit the actual ratio observed is typically significantly lower than the theoretical. Another approach relies on a sex mismatched experiment and using the signal ratio of the X chromosome to define the ratio for a single copy change (Fig. 5a). The drawback to this approach is that the ratio shift dampened by tissue heterogeneity is not reflected in the sex mismatch as both cancerous and non-cancerous cells in a sample have the same number of X chromosomes. Spectral karyotyping (SKY) or fluorescence *in situ* hybridization (FISH) can be used to calibrate the relationship between the copy number and the amplitude of the signal shift.

### Moving average based thresholding

In this method, thresholding is applied to multiple consecutive data points, rather than individual ones. This involves calculating the average across a sliding window of data points (e.g. 30 kb windows sliding at 10 kb intervals) (Fig. 5b). As such, larger-sized windows which incorporate more adjacent points would produce a smoother curve, but at a lower detection sensitivity. Conversely, smaller windows will detect the smaller alterations, but may introduce a higher number of false positives.

### K-means clustering

K-means clustering involves the *a priori* determination of a set of clusters, k, such that a given quantity is minimized relative to the centroids of the clusters (MacQueen, 1967). Moreover, the variability in the types of K-means clustering is achieved by changing the method of measuring distance and the quantity to be minimized. For example, one quantity to minimize is the maximum distance of an object to its centroid using a distance measure such as the Euclidean distance (Autio et al. 2003). In terms of array CGH, three centroids are normally used, to represent “gain”, “loss” and “retention” respectively. However, the number of centroids may be increased to reflect multiple levels of gains and losses.

### Hidden Markov model

Briefly, a Hidden Markov model (HMM) is a statistical approach designed for describing a system with unknown parameters using those that are observed—where the known aspects of the model are the states assigned and the unknown parts are the transition probabilities between states. Moreover, HMMs can be described by three main components: a set of probabilities associated with transitions between all states (Λ), a set of probability distributions associated with each state (B), and a distribution of initial states (π). Commonly, any HMM with a discrete, finite number of states can be defined as λ = (Λ, B, π) (Rabiner, 1989).

In the context of the application of HMM to array CGH analysis, a simple version of this approach was utilized where the hidden states in fact represented each of the states of copy number change; gain, loss and retention (de Vries et al. 2005). Moreover, this method has been used to extrapolate levels of copy number when accounting for such factors as tissue heterogeneity as the expected ratio change for a single copy gain and loss would be dampened (Fridlyand et al. 2004). In addition to the application to BAC based microarrays, this approach has been employed in the context of the oligonucleotide platforms (Iafrate et al. 2004; Nannya et al. 2005; Zhao et al. 2004).

### Circular binary segmentation

Circular binary segmentation (CBS) is a change-point based method which searches for particular change points where neighboring regions of DNA exhibit a statistical difference in copy number. By modifying the standard binary segmentation approach to a circular approach, this algorithm can be used to detect breakpoints in DNA as the altered region would be flanked by two regions of different copy number level, requiring two breakpoints. This algorithm, implemented in the *DNACopy* package, has been applied to test BAC array and representative oligonucleotide microarray (ROMA) datasets (Olshen et al. 2004). The application of CBS to describe genetic alterations myeloid sarcoma has been reported recently (Deeb et al. 2005).

### Wavelet-based

Another approach for array CGH analysis revolves around the use of wavelets. Briefly, this is a spatially-adaptive and non-parametric approach used to denoise (smooth) and segment data. Furthermore, this method can handle small discrete alterations which appear as an abrupt aberration and deal with the inherent property of variable sized alterations with different magnitudes seen in array CGH data (Hsu et al. 2005). This approach has been implemented in a few different algorithms used to smooth and segment array CGH data (Hsu et al. 2005; Khojasteh et al. 2006).

### Genetic local search

The genetic local search approach is an algorithm which tries to partition the data by placing a user-defined number of breakpoints across a particular chromosome. Breakpoints are placed in a random fashion and the algorithm iteratively tries to improve the location of the breakpoints such that the negative log-likelihood of the data and the penalty associated with too many breakpoints within a partition are minimized (Jong et al. 2004). Furthermore, the data becomes segmented and the values are “smoothed” such that they are the average of all the data points in that segment (Fig. 5c). This method, implemented in the *aCGH-Smooth* software package, has been used in the analysis of non-small cell lung cancer (NSCLC) cell lines (Garnis et al. 2006), small cell lung cancer (SCLC) cell lines (Coe et al. 2006), and oral squamous cell carcinoma (Baldwin et al. 2005).

### False discovery rate analysis and validation of copy number alterations

It should be noted that there is a false discovery rate (positive and negative) associated with any algorithm used for the detection of segmental alterations. The algorithm may not be able to consistently identify and correct for intrinsic noise in the data due to technical and biological variance encountered in array CGH experiments (Ylstra et al. 2006). Complementary methods such as fluorescence *in situ* hybridization and quantitative PCR will provide independent confirmation of the CGH findings. Alternatively, detection of changes in expression of genes within regions of alteration will also provide support of biological significance.

## Software Packages for Analysis and Visualization

Table 1 summarizes currently available array CGH software programs and compares the algorithms used in the detection of segmental copy number changes and the types of visualization available.

Typically, software programs are developed to support the analysis and/or visualization of specific array platforms, especially for the commercially available platforms. For example, Affymetrix (Affymetrix *Copy Number Analysis Tool*) and Nimblegen (Nimblegen *SignalMap*) have been developed by the respective companies for their manufactured arrays. In contrast, software applications developed by academic laboratories were generally designed to handle a primary array utilized by the research group and upon subsequent improvements, could handle data from other commonly used array platforms. The application *SeeGH*, as an example, was initially developed to visualize and analyze BAC array CGH data but in new versions of the application, data from oligonucleotide or cDNA platforms can be accommodated. Furthermore, other programs such as *ArrayCyGHt*, *CGH-Explorer*, *M-CGH* and *Normalise Suite v2.5* also demonstrate versatility by handling the data generated by all three types of array platforms (Table 1). The visualization capabilities of these applications are compared based on the ability to view single or multiple experiments, and simple static graphical representations versus interactive displays (Table 1). Here, we highlight three software examples to illustrate interactive display: *CGHPro*, *CGHAnalyzer v2.2* and *SeeGH v3.0*.

### CGHPro

*CGHPro* is a Java-based software operable on multiple operating systems. It requires the installation of the Java Runtime Environment Version 1.4.2 or higher, the statistical package R (Ihaka and Gentleman, 1996) Version 1.9.1 and the MySQL database server to store array CGH experiments (Chen et al. 2005). The major functionalities in this software include data quality assessment through graphical means, normalization of data using commonly used techniques for microarray imaging, integration of previously designed algorithms for alteration detection, and multiple methods for visualization. In addition, *CGHPro* can input formatted data from a variety of array platforms.

Data quality assessment is achieved using graphical methods such as scatter plots of the log_2_ spot intensities, box plots, histograms, M-A plots and QQ plots. Data filtering is achieved using user-defined parameters. Normalization routines include: Global Median, Subgrid Median, LOWESS (locally weighted scatter plot smooth), Subgrid LOWESS, and dye-swap normalization. Alteration detection algorithms include direct thresholding and thresholding after use of segmentation algorithms, incorporating the *aCGH bioconductor* (HMM) and *DNACopy* (CBS) packages (Fridlyand et al. 2004; Olshen et al. 2004). Visualization is interactive allowing sequential magnification and viewing of multiple experiments.

### CGHAnalyzer v2.2

*CGHAnalyzer* is also a Java-based software with the requirement of Java Runtime Environment version 1.4 or later (Margolin et al. 2005). This program allows querying of pre-loaded or custom gene sets for copy number status and integrates the clustering options of *TIGR Multi-Experiment Viewer* (Saeed et al. 2003). *CGHAnalyzer* does not have normalization functions requiring pre-normalized data. However, mapping information for UPenn BAC array and Affymetrix P501 SNP array are pre-loaded.

Two visualization layouts are provided to give the option of viewing the chromosomes in concentric circles or as traditional chromosome ideograms. Multiple experiments can be viewed using heatmap alignment of individual chromosomes. Alteration detection depends on direct thresholding or by variation from a pre-defined distribution.

### SeeGH v3.0

*SeeGH* was developed in C++, runs on Windows platform, requiring MySQL as the database structure. It accepts pre-normalized data and allows filtering of replica data points based on standard deviation and signal-to-noise ratio cut-offs. *SeeGH* accommodates data from a variety of sources, for example copy-number, gene expression, and global methylation profiles. Interactive display functions include sequential magnification, linking of clones to genes and, in turn, to biological databases (e.g. UCSC Genome Browser). Localization to specific regions of interest can be achieved through querying of identifiers such as gene name, clone name, and base pair position. Experimental parameters and user comments are stored within *SeeGH* allowing convenient information retrieval.

**Figure 6.**
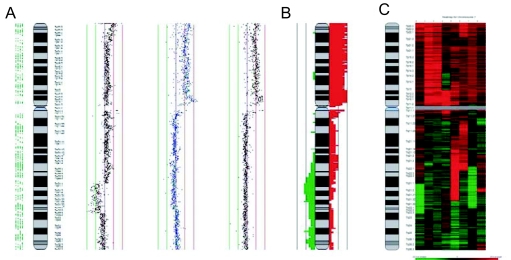
Examples of multiple experiment visualization methods in *SeeGH*. **A**: Multiple alignment of individual chromosome profiles. **B**: Frequency plot summarizing multiple experiments. Here, red histograms represent frequency of gains and green lost. **C**: Heatmap display of copy number status. Each vertical column represents an individual profile. Red indicates gain and green indicates loss. The amplitude of the ratio is reflected in the color intensity.

In addition, users can add customized or preloaded tracks to display gene location, CpG island position, microRNA location, etc. Multiple chromosome alignment, frequency summary plot, and heatmap display are included options for viewing multiple experiments (Fig. 6). Direct thresholding and moving average based thresholding are built in for alteration detection. Alternatively, segmentation using external software (e.g. *aCGH-Smooth*) can be imported for visualization.

### Considerations for future software development

With the rapid accumulation of large scale high throughput data describing cancer genomes, epigenomes, and transcriptomes, cross-platform meta-analysis will become prevalent. However, researchers with limited genomics and computational expertise will not be able to readily take advantage of such information. The development of facile, web-based software for the integration of large scale multidisciplinary databases will facilitate the widespread mining of genomic data and their correlation with clinical features (Kingsley et al. 2006). These issues are more pronounced with the increasing emphasis on translational research as array CGH technology moves towards clinical application. Added consideration of the ease of use, information security, automation and incorporation of prior knowledge of disease to assist in interpretation is necessary to deliver these emerging technologies to a clinical setting.

## Figures and Tables

**Table 1.** Software for analysis and visualization of array CGH data.

Software	Array Platform	Free/Cost	Computer Platform*	Alteration Detection	Display type‡	Profile Display	Website	Reference
*aCGH Analyzer v2.0*	cDNA	Free	W	No	G	Single	genomic.dfci.harvard.edu/array_cgh_tools.asp	-
*aCGH Smooth*	LIC, cDNA, oligo	Free	W	Heuristic algorithm, regularized maximum likelihood, Threshold	G	Single	www.few.vu.nl/~vumarray/	Jong et al. 2004
*aCGHViewer*	LIC, cDNA, oligo	Free	W, M, L, U	No	I	Multiple	falcon.roswellpark.org/aCGHview/	Shankar et al. 2006
*Affymetrix Copy Number Analysis Tool (CNAT)*	oligo	Free	W	Copy-number calculated based on SNP intensity of sample relative to distribution derived from 100 sample normal reference, Copy number response curve	I	Single	www.affymetrix.com/products/software/specific/cnat.affx	Huang et al. 2004
*ArrayCyGHt*	LIC, cDNA, oligo	Free	Web-based	Thresholding	G	Single	genomics.catholic.ac.kr/arrayCGH/	Kim et al. 2005
*Bioconductor’s aCGH package*	LIC	Free	W, M. L, U	Unsupervised Hidden Markov Partition	G	Single consensus plot	www.bioconductor.org and www.luc.ac.be/~jlindsey/rcode.html	Fridlyand et al. 2004
*Caryoscope v0.3.10*	viewer	Free	W, M, L, U, Web-based	Moving average, compute log ratio to any base, need GCOS and GTYPE	I	Single	caryoscope.stanford.edu/	Awad et al. 2004
*CGH Analytics v3.2*	cDNA, oligo	Cost, Free trial	W, M, L	Z-scoring, moving average calculation	I	Multiple	www.chem.agilent.com/Scripts/PDS.asp?lPage=29457	-
*CGHAnalyzer v2.2*	LIC, oligo	Free	W, M, U	Standard ratio threshold, p-value based on reference	I	Multiple	guanine.genomics.upenn.edu/people/faculty/weberb/downloads.htmCGH/html/	Margolin et al. 2005
*CGH-Explorer*	LIC, cDNA, oligo	Free	W, M, L	Thresholding, bootstrap-based method, Analysis of Copy Errors (ACE)	I	Multiple	www.ifi.uio.no/bioinf/Papers/CGH/	Lingjaerde et al. 2005
*CGH-Miner*	LIC, cDNA	Free	W, U	CLAC (clustering along chromosomes) with FDR (false discovery rate)	G	Single consensus plot	www-stat.stanford.edu/%7Ewp57/CGH-Miner/	Wang et al. 2005
*CGH-Plotter*	cDNA	Free	W, M, L	K-means clustering, dynamic programming	G	Multiple	sigwww.cs.tut.fi/TICSP/CGH-Plotter	Autio et al. 2003
*CGHPRO*	LIC, oligo	Free	W, L	Unsupervised Hidden Markov Partition, Circular Binary Segmentation	I	Multiple	molgen.mpg.de/~abt_rop/molecular_cytogenetics/ArrayCGH/CGHPRO	Chen et al. 2005
*ChARM v1.8*	cDNA	Free	W, M, L	Expectation Maximization (EM), one-sided sign test and/or mean permutation test	G	Multiple	function.princeton.edu/ChARM/	Myers et al. 2004
*CNAG*	oligo	Free	W	Hidden Markov model	I	Single	www.genome.umin.jp/	Nannya et al. 2005
*dCHIP*	cDNA, oligo	Free	W	Hidden Markov model, Median Smoothing, PM/MM Difference Model	I	Multiple	www.dchip.org	Zhao et al. 2004
*DIGMAP Viewer v1.15*	cDNA	Free	W	Clustering of cDNA expression data based on chromosome location	I	Multiple	geneexplorer.mc.vanderbilt.edu/digmap/	Yi et al. 2005
*DNACopy*	LIC, oligo	Free	W, M. L, U	Circular Binary Segmentation	G	Single	www.mskcc.org/mskcc/html/18551.cfm	Olshen et al. 2004
*GLAD*	LIC	Free	W, M. L, U	Adaptive Weights Smoothing	G	Single	Request author: glad@curie.fr	Hupe et al. 2004
*M-CGH*	LIC, cDNA, oligo	Free	W, M, L, U	Maximum likelihood and K-nearest neighbor or wavelet approach	I	Single	folk.uio.no/junbaiw/mcgh/	Wang et al. 2004
*Nimblegen SignalMap*	oligo	Cost	W	Windowed Threshold, Second Derivative Peak	I	Single	www.Nimblegen.com/products/software/signalmap.html	-
*Normalise Suite v2.5*	LIC, cDNA, oligo	Free	W	Region detection by user-defined thresholds or sliding window algorithm	I	Multiple	www.utoronto.ca/cancyto/index.html?protocols_software/software/index.html	Beheshti et al. 2003
*SeeGH v1.5*	LIC	Free	W	No	I	Single	www.bccrc.ca/arraycgh/SeeGH.htm	Chi et al. 2004
*SeeGH v3.0*	LIC, cDNA, oligo	Collab†	W	Moving average	I	Multiple	www.ArrayCGH.ca	-
*Spectral Ware v2.2*	LIC	Cost	Web-Based	Confidence interval, based on iterative algorithm	I	Single	www.spectralgenomics.com/spectralware.htm	-

* W, Windows; M, Macintosh; L, Linux; U, Unix.

‡ G, Graphical Representation; I, Interactive Display.

† Free on Collaborative basis.
